# Cardiovascular Biomarkers in Amniotic Fluid, Umbilical Arterial Blood, Umbilical Venous Blood, and Maternal Blood at Delivery, and Their Reference Values for Full-Term, Singleton, Cesarean Deliveries

**DOI:** 10.3389/fped.2019.00271

**Published:** 2019-07-02

**Authors:** Martin E. Blohm, Florian Arndt, Glenn M. Fröschle, Nora Langenbach, Jan Sandig, Eik Vettorazzi, Thomas S. Mir, Kurt Hecher, Jochen Weil, Rainer Kozlik-Feldmann, Stefan Blankenberg, Tanja Zeller, Dominique Singer

**Affiliations:** ^1^Division of Neonatology and Pediatric Intensive Care, Department of Pediatrics, University Children's Hospital, University Medical Center Hamburg-Eppendorf, Hamburg, Germany; ^2^Department of Pediatric Cardiology, University Heart & Vascular Center, University Medical Center Hamburg-Eppendorf, Hamburg, Germany; ^3^Center for Experimental Medicine, Institute of Medical Biometry and Epidemiology, University Medical Center Hamburg-Eppendorf, Hamburg, Germany; ^4^Department of Obstetrics and Fetal Medicine, University Medical Center Hamburg-Eppendorf, Hamburg, Germany; ^5^Department of General and Interventional Cardiology, University Heart & Vascular Center, University Medical Center Hamburg-Eppendorf, Hamburg, Germany; ^6^German Center for Cardiovascular Research, Partner Site Hamburg/Kiel/Lübeck, Hamburg, Germany

**Keywords:** pro-adrenomedullin, atrial natriuretic factor, atrial natriuretic peptide, copeptin, troponin I, neonate, human

## Abstract

**Background:** Several cardiovascular biomarkers have regulatory functions in perinatal physiology.

**Aim:** This study aimed to analyze the feto-maternal distribution pattern of biomarkers in samples of amniotic fluid, umbilical arterial blood, umbilical venous blood, and maternal blood samples, and to establish reference values. Each linked sample set consisted of the combined samples obtained in an individual pregnancy.

**Study design:** We performed a prospective, observational, cross-sectional, single-center study.

**Subjects:** The sample cohort included 189 neonates who were born to 170 mothers. A total of 162/189 neonates were full term and 129/189 were delivered by elective cesarean section.

**Outcome measures:** Midregional pro-adrenomedullin (MRproADM [nmol/L]), midregional pro-atrial natriuretic peptide (MRproANP [pmol/L]), brain natriuretic peptide (BNP [pg/mL]), N-terminal pro-brain natriuretic peptide (NTproBNP [pg/mL]), copeptin [pmol/L], and high-sensitive troponin I (hsTnI [pg/mL]) levels were measured.

**Results:** In singleton, full-term, primary cesarean deliveries (*n* = 91), biomarker levels (median, [IQR]) at delivery were as follows. MRproADM levels in umbilical arterial blood/umbilical venous blood/amniotic fluid/maternal blood were 0.88 (0.20)/0.95 (0.18)/2.80 (1.18)/1.10 (0.54), respectively. MRproANP levels were 214.23 (91.38)/216.03 (86.15)/0.00 (3.82)/50.67 (26.81), respectively. BNP levels were 14.60 (25.18)/22.08 (18.91)/7.15 (6.01)/6.20 (18.23), respectively. NTproBNP levels were 765.48 (555.24)/816.45 (675.71)/72.03 (55.58)/44.40 (43.94), respectively. Copeptin levels were 46.17 (290.42)/5.54 (9.08)/9.97 (7.44)/4.61 (4.59), respectively. Levels of hsTnI were 6.20 (4.25)/5.60 (5.01)/0.45 (1.73)/2.50 (2.40), respectively.

**Conclusion:** We determined reference values for biomarkers in term neonates delivered by primary cesarean section in amniotic fluid, umbilical arterial and venous blood, and maternal blood. Biomarkers in the fetal circulation appear to be of primary fetal origin, except for MRproADM.

## Introduction

A biomarker in medicine is a measurable parameter that can be used to assess a physiological or pathological state of an organism. Proteins in the blood stream can be used as biomarkers. Cardiovascular biomarkers are used to assess the cardiac and circulatory situation in adult medicine. These same biomarkers are also relevant in perinatal maternal and fetal physiology. Cardiovascular biomarkers can act as physiological regulators in the perinatal setting. Therefore, in the field of cardiovascular biomarkers, there is an interesting overlap between neonatology, obstetrics, and cardiology. Cardiovascular biomarkers may help to understand fetal and maternal physiology in pregnancy and during the perinatal transition period. Even though the fetal and maternal circulation is regulated independently in pregnancy and during delivery, an effect of fetal biomarkers on maternal physiology and vice versa has been postulated. Copeptin is a C-terminal component of pre-pro-arginine vasopressin and is secreted simultaneously with vasopressin. Vasopressin plays an important role in circulatory and stress regulation in maternal and fetal responses to labor (1, 2). Natriuretic peptides are of interest in the perinatal setting because they have important renal and cardiovascular effects during intrauterine and extrauterine life (3–7). During intrauterine life, the fetus needs to maintain sufficient urine production to provide amniotic fluid (AF). This is important for lung development. Natriuretic peptides are elevated in pregnancy (3) and may thus help to maintain sufficient fetal urine output. After birth, clearing fluid from the lungs to establish sufficient respiration is important. Vasopressin helps to clear pulmonary fluid (8). This indicates that biomarkers representing different physiological actions, such as natriuretic peptides and vasopressin/copeptin, are relevant for sufficient pulmonary function of newborns. Therefore, a combined analysis of several biomarkers (1–11) in the perinatal context could be useful for further determining perinatal/fetal and maternal physiology.

In adult medicine, cardiovascular biomarkers, such as N-terminal pro-brain natriuretic peptide (NTproBNP) and midregional pro-atrial natriuretic peptide (MRproANP), are mentioned in current guidelines on acute and chronic heart failure as diagnostic tools (12, 13). Large population-based studies combining several cardiovascular biomarkers (14, 15) are used as an approach to identify optimal cut-off values for individual biomarkers and diseases.

The authors of a recent editorial discussed the use of biomarkers in neonatology and expressed the need to establish reference values for biomarkers in different body fluids (16). This study aimed to analyze cardiovascular biomarkers in combined samples of amniotic fluid (AF), arterial umbilical blood (UAB), venous umbilical blood (UVB), and maternal blood (MATB) in the perinatal setting at the time of delivery. To the best of our knowledge, this is the first study providing a linked analysis of biomarkers in these four body fluids in individual pregnancies. The examined biomarkers were chosen on the basis of a previous study (17), and they are clinically used in adult medicine and relevant in the perinatal setting.

## Materials and Methods

### Study Design

The primary aim of the study was to measure and correlate cardiovascular biomarker levels between different fetal and maternal fluid compartments at the time of delivery. Secondary aims were to establish reference values and to identify factors affecting biomarker levels. The examined fluids were AF, UAB, UVB, and MATB. The examined biomarkers were midregional pro-adrenomedullin (MRproADM), MRproANP, brain natriuretic peptide (BNP), NTproBNP, copeptin, and high-sensitive troponin I (hsTnI). The study cohort was recruited from healthy term or late preterm pregnancies at the time of delivery. The study was conducted as a prospective, single-center, cross-sectional, observational study at the University Medical Center Hamburg-Eppendorf (UKE). Patients with consecutive term or late preterm pregnancies with delivery at the UKE, for whom antenatal agreement to participate in the study was obtained, were included. Patients with pregnancies with maternal conditions, such as assisted reproduction, gestational diabetes, HELLP syndrome (hemolysis, elevated liver enzymes, low platelets), and preeclampsia, were not excluded. Patients with pregnancies with fetal cardiac or chromosomal anomalies were excluded from the study. The study database did not provide maternal or fetal follow-up data. The study was approved by the local ethics committee of the Chamber of Physicians Hamburg, Germany. This study combined data with a previously published database (17). The present study tripled the sample size and added biomarker levels in UAB and AF.

### Patients

Matched samples of AF, UAB, UVB, and MATB were taken at the time of delivery with parental consent. Umbilical cord blood samples that were taken from the placenta represented perinatal, rather than purely fetal, blood samples because they reflected the status of the child at the time of cord clamping. For reasons of simplicity and to distinguish the mother and child, we chose the term “fetal” rather than “perinatal” throughout the text for cord blood samples. The study cohort included 189 neonates and their mothers (*n* = 170) (Table 1). Of the 189 neonates, 162 were full-term neonates, 129 were born by primary cesarean section, and 151 were singletons. There were 15 sets of twins and two sets of triplets. There were 93 male neonates. In the total cohort of 189 neonates, there were the following maternal conditions: four cases of HELLP (hemolysis, elevated liver enzymes, low platelets) or preeclampsia, 17 cases of maternal (gestational) diabetes (3/17 insulin-dependent), 11 cases of assisted reproduction, and five cases of oligohydramnious. A sample cohort subset of singleton, full-term, primary cesarean deliveries excluding one case of HELLP was chosen as the database for calculating reference values. In this group of 91 pregnancies (Table 2), there were 11 cases of maternal (gestational) diabetes (2/11 insulin dependent) and two cases of assisted reproduction.

**Table 1:** Sample characteristics for the total cross sectional study cohort (*n* = 189 neonates).

**Parameter**	**Mean**	**Median**	**SEM**	**SD**	**Minimum**	**Maximum**
Birth weight [kg]	3.191	3.280	0.051	0.707	0.807	5.305
Birth length [cm]	50.19	51.00	0.30	4.09	30.50	56.00
Head circumference [cm]	34.83	35.00	0.20	2.75	25.00	54.50
Apgar 1 min	8.78	9.00	0.07	0.92	3.00	10.00
Apgar 5 min	9.62	10.00	0.06	0.82	6.00	10.00
Apgar 10 min	9.85	10.00	0.04	0.49	7.00	10.00
Gestational age [weeks]	38.39	38.71	0.16	2.18	27.57	41.71
Umbilical arterial pH	7.30	7.32	0.01	0.08	7.03	7.44
Fetal arterial base excess [mmol/l]	−1.87	−0.70	0.29	3.89	−19.00	4.50
Maternal age [years]	33.70	34.00	0.36	4.99	21.00	46.00
Maternal weight [kg]	82.45	80.00	1.11	14.73	50.00	142.50

**Table 2:** Sample characteristics: singleton term neonates (*n* = 91 neonates) delivered by elective cesarean section used for reference value calculation.

**Parameter**	**Mean**	**Median**	**SEM**	**SD**	**Minimum**	**Maximum**
Birth weight [kg]	3.483	3.400	0.051	0.490	1.990	4.825
Birth length [cm]	51.52	51.00	0.23	2.17	45.50	56.00
Head circumference [cm]	35.41	35.00	0.15	1.44	30.50	39.50
Apgar 1 min	9.09	9.00	0.06	0.57	6.00	10.00
Apgar 5 min	9.82	10.00	0.06	0.55	7.00	10.00
Apgar 10 min	9.97	10.00	0.02	0.18	9.00	10.00
Gestational age [weeks]	38.86	38.86	0.06	0.60	37.00	41.00
Umbilical arterial pH	7.33	7.33	0.00	0.04	7.20	7.44
Fetal arterial base excess [mmol/l]	0.02	−0.10	0.20	1.88	−4.90	4.50
Maternal age [years]	34.41	36.00	0.52	4.93	22.00	43.00
Maternal weight [kg]	82.95	82.00	1.57	14.63	57.00	124.00

### Laboratory Measurements

Blood samples were processed within an average time of 2 h, centrifuged, aliquoted, and frozen at −80°C. Laboratory analyses for each biomarker were performed with duplicate analysis of stored samples using commercially available kits. MR-proADM levels were measured in EDTA plasma with a fluoroimmunoassay (BRAHMS MR-proADM KRYPTOR; BRAHMS GmbH, Hennigsdorf, Germany). The analytical limit of detection (LoD) of this assay was 0.08 nmol/L. The intra-assay coefficient of variation (CV) was 4.23% and the inter-assay CV was 10.49%. MRproANP levels were measured in EDTA plasma with a fluoroimmunoassay (BRAHMS MR-proANP KRYPTOR; BRAHMS GmbH) (LoD: 6.0 pmol/L, intra-assay CV: 4.76%, inter-assay CV: 2.39%). BNP levels were measured by immunoassay in serum samples (ARCHITECT i2000SR; Abbott Diagnostics, Abbott Park, IL, USA) (LoD: 10 pg/mL, intra-assay CV: 5.7%, inter-assay CV: 20.68%) (as stated by the manufacturer). NT-proBNP levels were measured in serum samples using the Elecsys proBNP II assay and the ELECSYS 2010 was used for detection (ECLIA; Roche Diagnostics, Mannheim, Germany) (LoD: 5 ng/L, intra-assay CV: 0.97 and 5.15% on two separate occasions, inter-assay CV: 5.86%). Copeptin was levels were measured in EDTA plasma with a fluoroimmunoassay (BRAHMS Copeptin us KRYPTOR; BRAHMS GmbH) (LoD: 4.8 pmol/L, intra-assay CV: 2.33%, inter-assay CV: 4.48%). Levels of hsTnI were assessed in serum samples (ARCHITECT STAT highly sensitive troponin I immunoassay; Abbott Diagnostics) (LoD: 1.9 ng/L, inter-assay CV: 2.23, 2.36, and 5.58% on separate occasions, inter-assay CV: 3.66%).

### Data Collection and Statistics

Demographic data and laboratory results were analyzed using SPSS software (SPSS 20.0® IBM, Armonk, NY, USA) and R software (R Foundation for Statistical Computing, version 3.5.2 [2018-12-20]; Vienna, Austria, https://www.R-project.org/). The mean, standard error of the mean (SEM), standard deviation (SD), median, and interquartile range (IQR) were calculated. Percentiles are shown for individual biomarkers and different body fluid components in full-term, singleton, cesarean deliveries. Nonparametric tests (Spearman's correlation, Wilcoxon rank test) were used as appropriate. The relationships of individual biomarkers within the different body fluid compartments in individual mother–child pairs and between the different biomarkers were analyzed and modeled. The effect of demographic factors (gestational age, delivery mode, and multiple pregnancies) was tested. Multivariate ANOVA analysis of the factors of biomarkers, source of biomarkers (i.e., different body fluids), and delivery mode was performed. ANOVA on the effect of maternal pathology (HELLP, preeclampsia, maternal [gestational] diabetes, and assisted reproduction) was performed. The significance level was set to *p1* < 0.05.

## Results

### Analysis of Factors Affecting Biomarker Levels

Multivariate ANOVA of biomarkers, the source of biomarkers, and delivery mode showed that these factors were all relevant and should thus be analyzed separately. Univariate ANOVA of the factors of maternal (gestational) diabetes and assisted reproduction did not show a significant effect on biomarker levels in the sample subgroup of full-term singletons born by elective cesarean section (chosen for reference value calculation) (Table 2). There was, however, an effect of “diabetes” on hsTnI in UAB (*p* < 0.01) and MRproADM in AF (*p* = 0.43). Univariate ANOVA of the factor HELLP was significant for all maternal biomarkers (*p* < 0.01), except for hsTnI (*p* = 0.08) and Copeptin (*p* = 0.92).

The effects of gestational age, labor, and multiple pregnancies on biomarker levels are shown in Table 3. The effect of gestational age on biomarker levels is shown in Supplements 1–6. Differences in the biomarkers between elective cesarean deliveries and deliveries by labor were observed. The effect of mode of delivery on the biomarker correlations is shown in Supplements 7, 8.

**Table 3:** Cardiovascular biomarkers in different body fluids in singleton term deliveries delivered by elective cesarean section.

**Biomarker**	**Body Fluid**	**Mean**	**SEM**	**Median**	**IQR**	**N**
MRproADM [nmol/L]	UAB	0.96	0.07	0.88	0.20	22
	UVB	0.97	0.02	0.95	0.18	65
	AF	2.84	0.20	2.80	1.18	37
	MATB	1.17	0.05	1.10	0.54	77
MRproANP [pmol/L]	UAB	221.72	10.83	214.23	91.38	48
	UVB	231.09	8.33	216.03	86.15	86
	AF	3.00	1.47	0.00	3.82	37
	MATB	55.37	2.74	50.67	26.81	87
BNP [pg/mL]	UAB	24.13	4.74	14.60	25.18	21
	UVB	27.59	3.58	22.08	18.91	66
	AF	8.34	0.92	7.15	6.01	38
	MATB	12.79	1.84	6.20	18.23	77
NTproBNP [pg/ml]	UAB	853.34	64.59	765.48	555.24	48
	UVB	979.43	60.95	816.45	675.71	86
	AF	84.74	7.06	72.03	55.58	37
	MATB	57.27	5.12	44.40	43.94	87
Copeptin [pmol/L]	UAB	188.90	44.41	46.17	290.42	46
	UVB	24.51	7.22	5.54	9.08	87
	AF	12.85	1.94	9.97	7.44	38
	MATB	8.46	1.25	4.61	4.59	86
HsTnI [pg/mL]	UAB	16.44	6.91	6.20	4.25	45
	UVB	14.30	4.84	5.60	5.01	86
	AF	1.13	0.24	0.45	1.73	38
	MATB	3.08	0.24	2.50	2.40	87

*UVB, umbilical venous blood; UAB, umbilical arterial blood; AF, amniotic fluid; MATB, maternal blood. The database consisted of n = 91 neonates and their mothers*.

### Comparison of Biomarker Levels in Different Body Fluids

**Figure 1:**
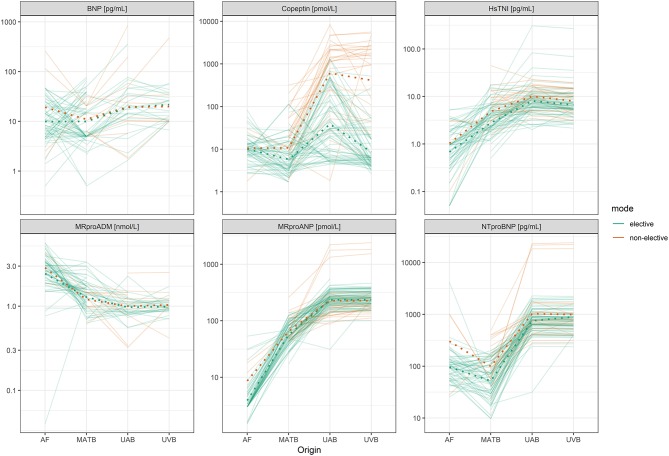
Graphs showing the measured biomarkers in amniotic fluid (AF), maternal blood (MATB), umbilical arterial blood (UAB), and umbilical venous blood (UVB). The individual color coded lines represent individual combined linked sample sets related to individual deliveries. Each line represents linked biomarker levels in an individual pregnancy. Green lines (elective) represent linked samples resulting from elective cesarean section. Orange lines (non-elective) represent non-elective deliveries with exposure to labor (i.e., vaginal delivery and secondary cesarean section). The database consisted of 189 neonates and their mothers.

**Figure 2:**
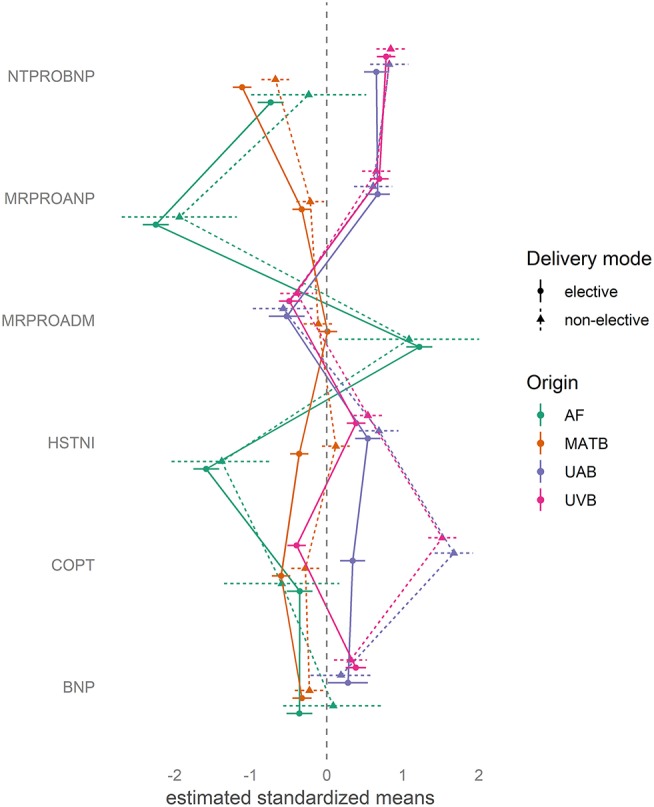
Distribution of biomarkers in color coded body fluid components of amniotic fluid (AF), maternal blood (MATB), umbilical arterial blood (UAB), and umbilical venous blood (UVB). Data are expressed as the standardized mean and SEM of the different biomarkers in relation to an estimated modeled mean. Data distinguish between elective and non-elective deliveries. Solid lines and dots represent elective cesarean deliveries (elective). Dashed lines and triangles represent deliveries with exposure to labor (i.e., vaginal delivery or secondary cesarean section, [non-elective]). The database consisted of 189 neonates and their mothers.

The distribution of biomarkers in the fluid compartments of AF, MATB, UAB, and UVB is shown in Figure 1. Figure 2 shows estimated mean relative biomarker levels in the different body fluids for each analyzed biomarker. The highest levels of MRproADM were observed in AF, followed by MATB, and the lowest levels were in fetal blood. Levels of the natriuretic peptides MRproANP, BNP, and NTproBNP were higher in fetal than in MATB. Copeptin levels in UAB were higher than those in UVB. All biomarkers (except MRproADM) were found in higher concentrations in fetal blood than in MATB.

Bivariate Spearman's correlations for the six examined biomarkers (MRproADM, MRproANP, BNP, NTproBNP, copeptin, hsTnI) were examined by comparing their levels between the different body fluids. Detailed data are presented for each studied biomarker in Supplements 1–6 as a correlation matrix.

**Figure 3:**
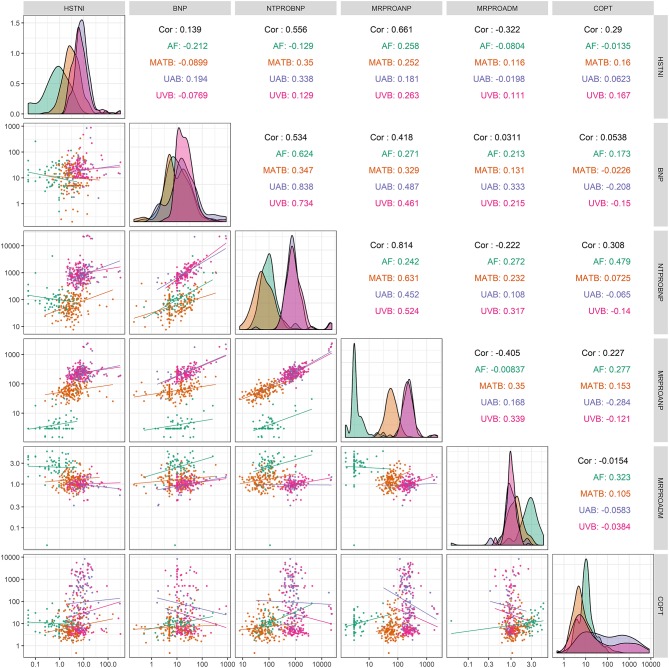
Correlation matrix shows bivariate correlations between the six analyzed biomarkers in four different body fluids as scatterplots and correlation coefficients (Spearman's rho). The superimposed curves shown in the diagonal center axis represent frequency curves for individual values in the individual body fluids. The four examined body fluids are color coded as follows: amniotic fluid (AF), green; maternal blood (MATB), orange; umbilical arterial blood (UAB), blue; and umbilical venous blood (UVB), purple. The examined biomarkers were high-sensitive troponin I (hsTnI [pg/mL]), brain natriuretic peptide (BNP [pg/mL]), N-terminal pro-brain natriuretic peptide (NTproBNP [pg/mL]), midregional pro-atrial natriuretic peptide (MRproANP [pmol/L]), midregional pro-adrenomedullin (MRproADM [nmol/L]), and copeptin (COPT [pmol/L]). The database consisted of 189 neonates and their mothers.

Bivariate correlations between the various biomarkers in the different body fluids for the total cohort are shown in Figure 3. Significant biomarker correlations in AF were: BNP-NTproBNP (*p* < 0.01), Copeptin-NTproBNP (*p* < 0.01), Copeptin-MRproADM (*p* = 0.01), Copeptin-MRproANP (*p* = 0.03), BNP-MRproANP (*p* = 0.03), MRproADM-NTproBNP (*p* = 0.03), hsTnI-MRproANP (*p* = 0.04). In MATB significant correlations were: MRproANP-NTproBNP (*p* < 0.01), hsTnI-NTproBNP (*p* < 0.01), MRproADM-MRproANP (*p* < 0.01), BNP-NTproBNP (*p* < 0.01), BNP-MRproANP (*p* < 0.01), hsTnI-MRproANP (*p* < 0.01), MRproADM-NTproBNP (*p* < 0.01), Copeptin-hsTnI (*p* = 0.03), Copeptin-MRproANP (*p* = 0.42). In UAB significant correlations were: BNP-NTproBNP (*p* < 0.01), MRprANP-NTproBNP (*p* < 0.01), hsTnI-NTproBNP (*p* < 0.01), BNP-MRproANP (*p* < 0.01), Copeptin-MRproANP (*p* < 0.01), BNP-MRproADM (*p* < 0.05). In UVB significant correlations were: BNP-NTproBNP (*p* < 0.01), MRprANP-NTproBNP (*p* < 0.01), BNP-MRproANP (*p* < 0.01), MRprADM-MRproANP (*p* < 0.01), NTproBNP-MRproADM (*p* < 0.01), hsTnI-MRproANP (*p* < 0.01), BNP-MRproADM (*p* = 0.01), Copeptin-hsTnI (*p* = 0.03).

### Biomarker Levels and Reference Values in Primary Cesarean Deliveries of Full-Term Singletons

Biomarker levels and reference values are shown in Table 3. Reference values at the time of delivery are provided as percentiles in Table 4.

**Table 4:** Percentiles for cardiovascular biomarkers in different body fluids in term singleton deliveries.

**Biomarker**	**Fluid**	**Percentiles**							
		**5**	**10**	**25**	**50**	**75**	**90**	**95**	**N**
MRproADM [nmol/L]	UAB	0.56	0.63	0.82	0.88	1.02	1.13	2.11	22
	UVB	0.71	0.76	0.86	0.95	1.05	1.18	1.36	65
	AF	0.69	0.93	2.32	2.80	3.50	4.51	5.02	37
	MATB	0.65	0.74	0.88	1.10	1.42	1.62	2.17	77
MRproANP [pmol/L]	UAB	113.55	142.19	170.53	214.23	261.90	336.66	375.04	48
	UVB	144.01	151.06	173.66	216.03	259.81	340.04	387.39	86
	AF	0.00	0.00	0.00	0.00	3.82	6.88	13.13	37
	MATB	24.48	27.99	38.86	50.67	65.67	90.21	108.20	87
BNP [pg/mL]	UAB	1.72	2.74	10.08	14.60	35.25	66.87	75.98	21
	UVB	10.00	10.00	12.65	22.08	31.56	41.32	76.85	66
	AF	1.36	2.87	4.56	7.15	10.58	14.67	25.09	38
	MATB	0.00	0.00	0.00	6.20	18.23	34.53	48.31	77
NTproBNP [pg/mL]	UAB	259.84	388.89	547.39	765.48	1102.63	1515.55	1819.50	48
	UVB	369.62	397.81	597.91	816.45	1273.63	1743.40	2233.15	86
	AF	31.54	41.01	52.92	72.03	108.50	129.04	212.10	37
	MATB	11.92	17.49	26.95	44.40	70.89	130.03	149.75	87
Copeptin [pmol/L]	UAB	5.02	5.49	12.34	46.17	302.76	500.00	1094.72	46
	UVB	3.06	3.43	3.93	5.54	13.02	53.25	138.73	87
	AF	2.79	2.99	6.12	9.97	13.55	31.44	46.75	38
	MATB	1.68	2.18	3.17	4.61	7.76	20.18	38.64	86
HsTnI [pg/mL]	UAB	2.36	2.60	4.98	6.20	9.23	23.66	69.89	45
	UVB	2.15	2.65	3.58	5.60	8.59	13.61	31.72	86
	AF	0.00	0.00	0.09	0.45	1.81	3.54	5.16	38
	MATB	0.61	0.99	1.55	2.50	3.95	5.82	7.09	87

*UVB, umbilical venous blood; UAB, umbilical arterial blood; AF, amniotic fluid; MATB, maternal blood. The database consisted of n = 91 neonates and their mothers*.

## Discussion

The main novelty of this study was combined analysis of linked biomarker sample sets from individual pregnancies in the mother and child at the time of delivery, including AF, UAB, UVB, and MATB. Additionally, we calculated reference values for singleton, full-term cesarean deliveries for six different biomarkers (MRproADM, MRproANP, BNP, NTproBNP, copeptin, hsTnI) in these four different body fluids.

Theoretical considerations on production and clearance of these biomarkers may be derived using the simultaneous distribution pattern of individual biomarkers over different fetal and maternal fluid compartments. The highest levels of MRproADM were observed in AF, followed by MATB, and the lowest levels were in fetal blood. Additionally, MRproADM levels were positively correlated with UVB and UAB. This biomarker pattern may support a placental source of this biomarker. Alternatively, fetal renal excretion into the AF might have occurred. Higher MRproADM levels in multiple pregnancies with a relatively larger amount of total placental tissue, as shown in our study (Table 5), and lower levels in placental insufficiency (18) support a placental origin of MRproADM.

**Table 5:** Factors influencing biomarker levels: gestational age, labor, multiple gestation (Spearman's correlation, rho).

**Biomarker**	**Fluid**	**Gestational age**	**Exposure to labor**	**Multiple gestation**	**N**
MRproADM	UAB	−0.548^**^	0.172	0.378^*^	42
	UVB	−0.328^**^	0.180^*^	0.126	134
	AF	0.048	0.040	−0.194	63
	MATB	−0.126	0.010	0.383^**^	145
MRproANP	UAB	−0.555^**^	−0.207^*^	0.453^**^	98
	UVB	−0.453^**^	−0.205^**^	0.334^**^	173
	AF	−0.278^*^	0.166	0.291^*^	64
	MATB	−0.015	0.180^*^	0.199^*^	160
BNP	UAB	−0.479^**^	−0.073	0.497^**^	36
	UVB	−0.163	−0.160	0.063	137
	AF	−0.484^**^	0.020	0.526^**^	66
	MATB	0.074	0.080	0.100	145
NTproBNP	UAB	−0.289^**^	0.025	0.057	97
	UVB	−0.320^**^	−0.049	0.106	174
	AF	−0.460^**^	0.118	0.385^**^	64
	MATB	0.044	0.395^**^	0.116	160
Copeptin	UAB	0.460^**^	0.561^**^	−0.401^**^	93
	UVB	0.260^**^	0.653^**^	−0.137	175
	AF	−0.237	0.070	0.149	65
	MATB	0.131	0.251^**^	0.057	159
hsTnI	UAB	−0.177	0.244^*^	0.135	91
	UVB	−0.136	0.158^*^	0.081	174
	AF	0.081	0.155	−0.080	66
	MATB	0.025	0.294^**^	0.052	160

UVB, umbilical venous blood; UAB, umbilical arterial blood; AF, amniotic fluid; MATB, maternal blood; Spearman's correlation, rho. Significance level of correlation:

* *p < 0.05*,

** *p < 001. The database consisted of n = 189 neonates and their mothers*.

Levels of the natriuretic peptides MRproANP, BNP, and NTproBNP were higher in fetal than in MATB (Figures 1, 2). In paired UVB and UAB samples, natriuretic peptides (MRproANP, BNP, and NTproBNP) were correlated (Figure 3). Levels of MRproANP and NTproBNP were higher in UVB than in UAB (Figure 2). This distribution of natriuretic peptides suggests fetal production of the precursor molecules pro-BNP (7) and pro-ANP (19, 20), and subsequent placental cleavage of MRproANP and NTproBNP from the precursors. These findings support the hypothesis that the fetus is the main source of natriuretic peptides in the fetal circulation. There was an inverse correlation between fetal natriuretic peptide levels and gestational age (Table 5, Supplements 1–6), consistent with our previous study (17). The fetal circulation and AF production in midgestation appear to require physiologically elevated natriuretic peptide levels. These fetal natriuretic peptide levels are as high as those in adults with New York Heart Association class IV cardiac insufficiency (12, 13).

In our study, copeptin appeared to be produced by the fetus during labor. Copeptin levels in UAB were higher than those in UVB. Neonates who were exposed to labor (Table 5) had much higher copeptin levels than those born by elective cesarean section. In our study, fetal copeptin levels were higher than maternal copeptin levels. Interestingly, previous studies on copeptin have shown higher copeptin levels in the fetus than in the mother in secondary cesarean sections performed for fetal distress (21, 22). Furthermore, copeptin levels are lower in the fetus than in the mother in cesarean sections performed for reasons other than fetal distress (21, 22). Copeptin may be used as an indicator for fetal distress (21–23).

The distribution pattern for hsTnI appeared compatible with placental biomarker clearance between UAB and UVB and/or a transfer of hsTnI to MATB (Figures 2, 3).

Biomarkers appear to be regulated independently in the fetal and maternal circulations. Except for NTproBNP, all examined biomarkers showed a weak positive correlation between UVB and MATB (Supplements 1–6). If a biomarker is present at higher levels in the fetus than in the mother and there is a positive feto-maternal correlation, feto-maternal transfer of that biomarker may occur. Alternatively, a feto-maternal correlation might be caused by placental or hormonal effects on the mother and child, affecting biomarker levels in the same direction.

We calculated reference values for biomarkers in healthy term pregnancies in AF, UAB, UVB, and MATB. In addition to previous literature data on the examined biomarkers (1–11, 18–26), this study combined neonatal and maternal biomarker levels in different body fluids, including AF. We did not examine the prognostic relevance of individual biomarkers (23, 27). The factors of gestational age, multiple pregnancies, and exposure to labor did affect biomarker levels (Table 5). Therefore, we limited reference value calculation to full-term singletons who were delivered by elective cesarean section.

There are several potential limitations in our study. Because of the necessity to obtain informed consent for sample collection before delivery, neonates who were delivered by elective cesarean section are over-represented in the studied sample cohort (68%) compared with the local cesarean section rate of <30%. AF can easily be obtained in elective cesarean section, but is difficult to obtain during normal vaginal delivery. Therefore, we chose to provide reference values for the well-defined subgroup of healthy, full-term, singleton, elective cesarean deliveries to avoid bias by delivery mode, gestational age, and multiple pregnancies.

Samples were collected and analyzed in several batches. This might have caused analytical bias between separate occasions of measurement. The commercially available kits that we used have not been tested for analyzing AF, which might have caused methodical bias. In several BNP samples, the lower limit of the analysis was set to 10 units (see Table 3), as recommended by the manufacturer. This limit aggravated the skewness of distribution of BNP levels. The timing of maternal blood samples (taken before or after delivery), and thus the effect of labor (1, 2) or intravenous hydration during cesarean section, on maternal biomarker levels may have affected maternal biomarker levels. Despite this bias, our data support a general difference between maternal and fetal biomarker levels. The amount of blood and AF that were obtained was not sufficient to analyze all examined biomarkers in all feto-maternal matched samples, which resulted in a reduction in sample size. Because unclamped umbilical blood flow may continue several minutes after delivery (28–30), variation in delayed umbilical cord clamping might affect fetal biomarker levels.

## Conclusion

The main novelty of this study was combined analysis of linked biomarker sample sets of four different body fluids in healthy pregnancies at the time of delivery. Levels of six different cardiovascular biomarkers were measured, including MRproADM, MRproANP, BNP, NTproBNP, copeptin, and hsTnI. The observed biomarker distribution patterns suggest that most cardiovascular biomarkers in the fetal circulation are of primary fetal origin. Biomarker reference values for singleton, full-term, cesarean deliveries are provided.

## Data Availability

The datasets generated for this study are available on request to the corresponding author.

## Ethics Statement

This was purely an observational study. The study was carried out in accordance with the recommendations of the Ethics Committee of the Chamber of Physicians Hamburg and the Declaration of Helsinki. Written informed consent was obtained in accordance with the Declaration of Helsinki from all subjects. The protocol was approved by the local Chamber of Physicians, Hamburg, Germany.

## Author Contributions

MB, FA, JS, DS, and JW designed the study. MB, GF, NL, JS, and FA were involved in sample collection and data analysis. EV provided statistical advice, analyzed the data, and developed models to describe the data. MB, FA, GF, NL, JS, TM, KH, JW, RK-F, and DS contributed to data analysis and data interpretation. TZ, GF, NL, JS, and SB were involved in sample processing and laboratory analysis. JS, GF, and NL participated in the project as part of their medical theses. JS (31) and GF (32) have published their medical thesis (they analyzed different and additional aspects of parts of the data). MB prepared the manuscript. All authors read and approved the manuscript.

## Contribution to the Field Statement

A biomarker in medicine is a measurable parameter that can be used to assess the physiological or pathological state of an organism. Several proteins in the blood stream can be used as biomarkers. Cardiovascular biomarkers are used to assess the cardiac and circulatory situation in adult medicine. These biomarkers are also relevant in perinatal maternal and fetal physiology. Therefore, in the field of cardiovascular biomarkers, there is an interesting overlap between neonatology, obstetrics, and cardiology. This study analyzed six different cardiovascular biomarkers (derivate of adrenomedullin, three different natriuretic peptides, copeptin, and troponin) in four different body fluids (amniotic fluid, umbilical arterial and umbilical venous blood, and maternal blood) at the time of delivery. The distribution pattern of these biomarkers between maternal and fetal fluids is described. Reference values for the different biomarkers in singleton, full-term pregnancies are provided for these biomarkers in different body fluids. Most biomarkers showed higher levels in fetal than in maternal blood. Our data suggest that the source of these biomarkers in the fetal circulation is the fetus. Adrenomedullin, which was highest in amniotic fluid, might originate from a placental source or from fetal urine, which is the main source of amniotic fluid.

### Conflict of Interest Statement

The authors declare that the research was conducted in the absence of any commercial or financial relationships that could be construed as a potential conflict of interest.

## Abbreviations

Body fluids: AF: amniotic fluid
MATB: maternal blood
UAB: umbilical arterial blood
UVB: umbilical venous blood
Biomarker: BNP: brain natriuretic peptide
hsTnI: high-sensitive troponin I
MRproADM: midregional pro-adrenomedullin
MRproANP: midregional pro-atrial natriuretic peptide
NTproBNP: N-terminal pro-brain natriuretic peptide
Other abbreviations: CV: coefficient of variation
HELLP syndrome: hemolysis, elevated liver enzymes low platelets
LoD: limit of detection.

## References

[1] Wellmann S Koslowski A Spanaus K Zimmermann R Burkhardt T. Fetal release of copeptin in response to maternal oxytocin administration: a randomized controlled trial. Obstet Gynecol. (2016) 128:699–703. 10.1097/AOG.0000000000001594 27607861

[2] Evers KS Wellmann S. Arginine vasopressin and copeptin in perinatology. Front Pediatr. (2016) 4:75. 10.3389/fped.2016.00075 27532032 PMC4969663

[3] Mannarino S Garofoli F Mongini E Cerbo RM Codazzi AC Tzialla C. BNP concentrations and cardiovascular adaptation in preterm and fullterm newborn infants. Early Hum Dev. (2010) 86:295–8. 10.1016/j.earlhumdev.2010.04.003 20488634

[4] El-Khuffash A Molloy EJ. Are B-type natriuretic peptide (BNP) and N-terminal-pro-BNP useful in neonates? Arch Dis Child Fetal Neonatal Ed. (2007) 92:F320–4. 10.1136/adc.2006.106039 17585100 PMC2675431

[5] Kulkarni M Gokulakrishnan G Price J Fernandes CJ Leeflang M Pammi M. Diagnosing significant PDA using natriuretic peptides in preterm neonates: a systematic review. Pediatrics. (2015) 135:e510–25. 10.1542/peds.2014-1995 25601976

[6] Nir A Lindinger A Rauh M Bar-Oz B Laer S Schwachtgen L. NT-pro-B-type natriuretic peptide in infants and children: reference values based on combined data from four studies. Pediatr Cardiol. (2009) 30:3–8. 10.1007/s00246-008-9258-4 18600369

[7] Weisz DE McNamara PJ El-Khuffash A. Cardiac biomarkers and haemodynamically significant patent ductus arteriosus in preterm infants. Early Hum Dev. (2017) 105:41–7. 10.1016/j.earlhumdev.2016.12.007 27998626

[8] Cummings JJ Carlton DP Poulain FR Fike CD Keil LC Bland RD. Vasopressin effects on lung liquid volume in fetal sheep. Pediatr Res. (1995) 38:30–5. 10.1203/00006450-199507000-00006 7478793

[9] Grass B Baumann P Arlettaz R Fouzas S Meyer P Spanaus K. Cardiovascular biomarkers pro-atrial natriuretic peptide and pro-endothelin-1 to monitor ductus arteriosus evolution in very preterm infants. Early Hum Dev. (2014) 90:293–8. 10.1016/j.earlhumdev.2014.03.002 24661445

[10] Vijlbrief DC Benders MJ Kemperman H van Bel F de Vries WB. Use of cardiac biomarkers in neonatology. Pediatr Res. (2012) 72:337–43. 10.1038/pr.2012.88 22797141

[11] Admaty D Benzing J Burkhardt T Lapaire O Hegi L Szinnai G. Plasma midregional proadrenomedullin in newborn infants: impact of prematurity and perinatal infection. Pediatr Res. (2012) 72:70–6. 10.1038/pr.2012.38 22447319

[12] McMurray JJ Adamopoulos S Anker SD Auricchio A Böhm M Dickstein K. ESC Guidelines for the diagnosis and treatment of acute and chronic heart failure 2012: The Task Force for the Diagnosis and Treatment of Acute and Chronic Heart Failure 2012 of the European Society of Cardiology. Developed in collaboration with the Heart Failure Association (HFA) of the ESC. Eur Heart J. (2012) 33:1787–847. 10.1093/eurjhf/hft016 22611136

[13] Roberts E Ludman AJ Dworzynski K Al-Mohammad A Cowie MR McMurray JJ. The diagnostic accuracy of the natriuretic peptides in heart failure: systematic review and diagnostic meta-analysis in the acute care setting. BMJ. (2015) 350:h910. 10.1136/bmj.h910 25740799 PMC4353288

[14] Holm H Nägga K Nilsson ED Ricci F Melander O Hansson O. Biomarkers of microvascular endothelial dysfunction predict incident dementia: a population-based prospective study. J Intern Med. (2017) 282:94–101. 10.1111/joim.12621 28407377

[15] Schnabel RB Wild PS Wilde S Ojeda FM Schulz A Zeller T. Multiple biomarkers and atrial fibrillation in the general population. PLoS ONE. (2014) 9:e112486. 10.1371/journal.pone.0112486 25401728 PMC4234420

[16] Meyer S Zemlin M Poryo M. Editorial: biomarkers in neonatology. Early Hum Dev. (2017) 105:23–24. 10.1016/j.earlhumdev.2016.12.001 28041646

[17] Blohm ME Arndt F Sandig J Diehl W Zeller T Mueller GC. Cardiovascular biomarkers in paired maternal and umbilical cord blood samples at term and near term delivery. Early Hum Dev. (2016) 94:7–12. 10.1016/j.earlhumdev.2016.01.001 26851448

[18] Matson BC Corty RW Karpinich NO Murtha AP Valdar W Grotegut CA. Midregional pro-adrenomedullin plasma concentrations are blunted in severe preeclampsia. Placenta. (2014) 35:780–3. 10.1016/j.placenta.2014.07.003 25043691 PMC4143458

[19] Vesely DL. Atrial natriuretic peptide prohormone gene expression: hormones and diseases that upregulate its expression. IUBMB Life. (2002) 53:153–9. 10.1080/15216540212336 12102171

[20] Morgenthaler NG Struck J Thomas B Bergmann A. Immunoluminometric assay for the midregion of pro-atrial natriuretic peptide in human plasma. Clin Chem. (2004) 50:234–6. 10.1373/clinchem.2003.021204 14709661

[21] Burkhardt T Schwabe S Morgenthaler NG Natalucci G Zimmermann R Wellmann S. Copeptin: a marker for stress reaction in fetuses with intrauterine growth restriction. Am J Obstet Gynecol. (2012) 207:497.e1–5. 10.1016/j.ajog.2012.09.024 23089587

[22] Foda AA Abdel Aal IA. Maternal and neonatal copeptin levels at cesarean section and vaginal delivery. Eur J Obstet Gynecol Reprod Biol. (2012) 165:215–8. 10.1016/j.ejogrb.2012.08.012 22921847

[23] Schlapbach LJ Frey S Bigler S Manh-Nhi C Aebi C Nelle M. Copeptin concentration in cord blood in infants with early-onset sepsis, chorioamnionitis and perinatal asphyxia. BMC Pediatr. (2011) 11:38. 10.1186/1471-2431-11-38 21595972 PMC3118890

[24] Koch A Singer H. Normal values of B type natriuretic peptide in infants, children, and adolescents. Heart. (2003) 89:875–8. 10.1136/heart.89.8.875 12860862 PMC1767791

[25] Mannarino S Garofoli F Cerbo RM Perotti G Mongini E Codazzi C. Cord blood, perinatal BNP values in term and preterm newborns. Arch Dis Child Fetal Neonatal Ed. (2010) 95:F74. 10.1136/adc.2009.158683 20019201

[26] Koch L Dabek MT Frommhold D Poeschl J. Stable precursor fragments of vasoactive peptides in umbilical cord blood of term and preterm infants. Horm Res Paediatr. (2011) 76:234–9. 10.1159/000329285 21893934

[27] Kelen D Andorka C Szabó M Alafuzoff A Kaila K Summanen M. Serum copeptin and neuron specific enolase are markers of neonatal distress and long-term neurodevelopmental outcome. PLoS ONE. (2017) 12:e0184593. 10.1371/journal.pone.0184593 28931055 PMC5607206

[28] Finnemore A Groves A. Physiology of the fetal and transitional circulation. Semin Fetal Neonatal Med. (2015) 20:210–6. 10.1016/j.siny.2015.04.003 25921445

[29] Yao AC Hirvensalo M Lind J. Placental transfusion-rate and uterine contraction. Lancet. (1968) 1:380–3. 10.1016/S0140-6736(68)91352-4 4169972

[30] Boere I Roest AA Wallace E Ten Harkel AD Haak MC Morley CJ. Umbilical blood flow patterns directly after birth before delayed cord clamping. Arch Dis Child Fetal Neonatal Ed. (2015) 100:F121–5. 10.1136/archdischild-2014-307144 25389141

[31] Sandig J Kardiale biomarker und impedanzkardiographie in der neonatologie (Dissertation). Hamburg, Germany (2017). Available online at: http://ediss.sub.uni-hamburg.de/volltexte/2017/8780/pdf/Dissertation.pdf

[32] Fröschle GM Biomarkerspiegel in der perinatalmedizin: Normwerterstellung und vergleich von maternalen und kindlichen spiegeln von hsTnI, BNP, NT-proBNP, MR-proADM, MR-proANP und copeptin in der nabelvene, der nabelarterie, im fruchtwasser und im blut der mutter (Dissertation). Hamburg, Germany (2017).

