# Supplementary material for: Cardiovascular Biomarkers in Amniotic Fluid, Umbilical Arterial Blood, Umbilical Venous Blood, and Maternal Blood at Delivery, and Their Reference Values for Full-Term, Singleton, Cesarean Deliveries
Source: Front Pediatr. 2019 Jul 2;7:271. doi: 10.3389/fped.2019.00271 (PMC6614192; doi:10.3389/fped.2019.00271)
Supplement: Supplementary file 3 [file Table_3.DOCX]

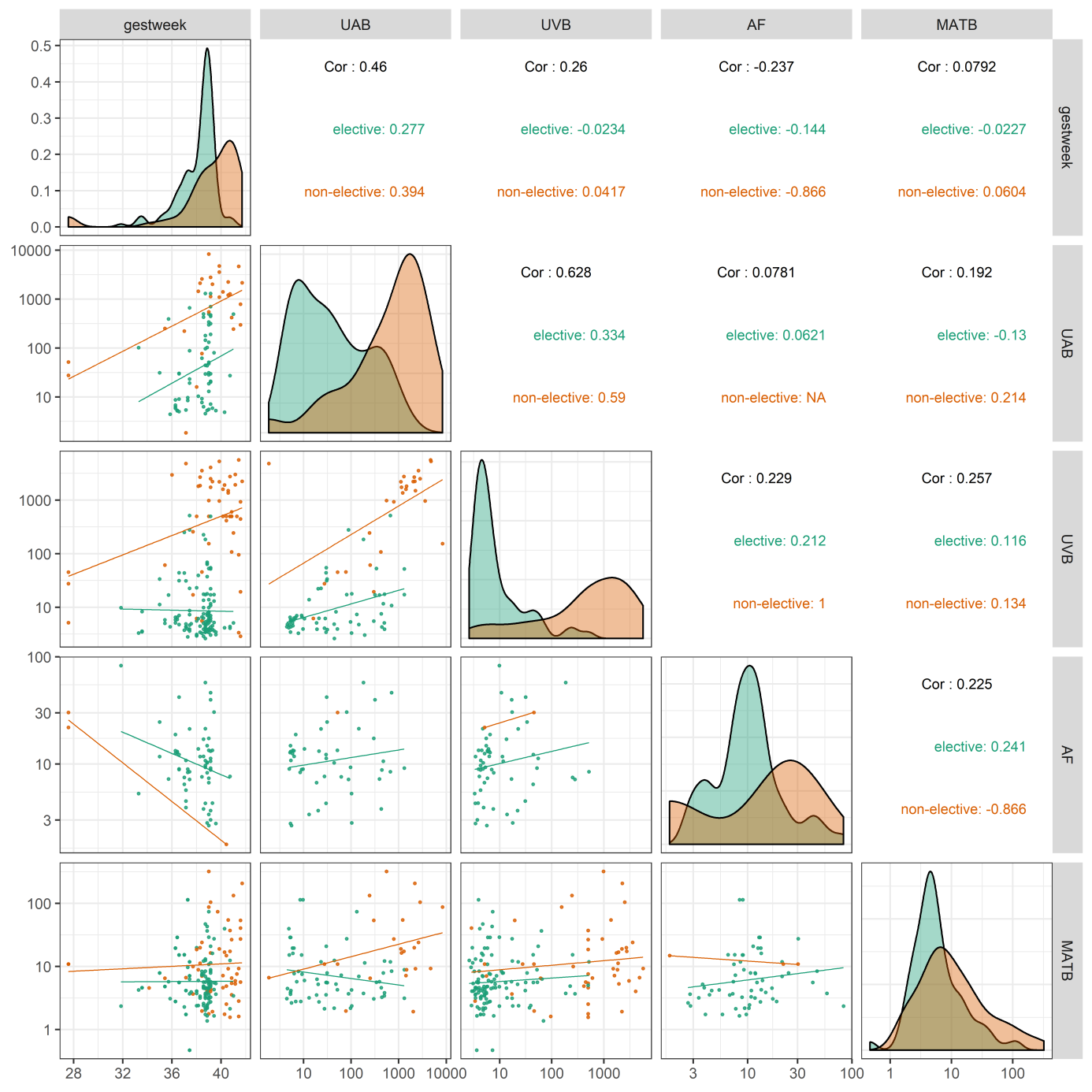


Supplement 3: Correlation matrix for HsTNI [pg/mL]. Bivariate correlations between four different body fluids and gestational age as scatterplots and correlation coefficients (Spearman's rho) are shown. The superimposed curves shown in the diagonal center axis represent frequency curves for individual values in the individual body fluids. The delivery mode is color coded (green for elective cesarean section, orange for non-elective delivery, i. e. for normal vaginal delivery or secondary cesarean section). The database consisted of n=189 neonates and their mothers.
