# Supplementary material for: Cardiovascular Biomarkers in Amniotic Fluid, Umbilical Arterial Blood, Umbilical Venous Blood, and Maternal Blood at Delivery, and Their Reference Values for Full-Term, Singleton, Cesarean Deliveries
Source: Front Pediatr. 2019 Jul 2;7:271. doi: 10.3389/fped.2019.00271 (PMC6614192; doi:10.3389/fped.2019.00271)
Supplement: Supplementary file 7 [file Table_7.DOCX]

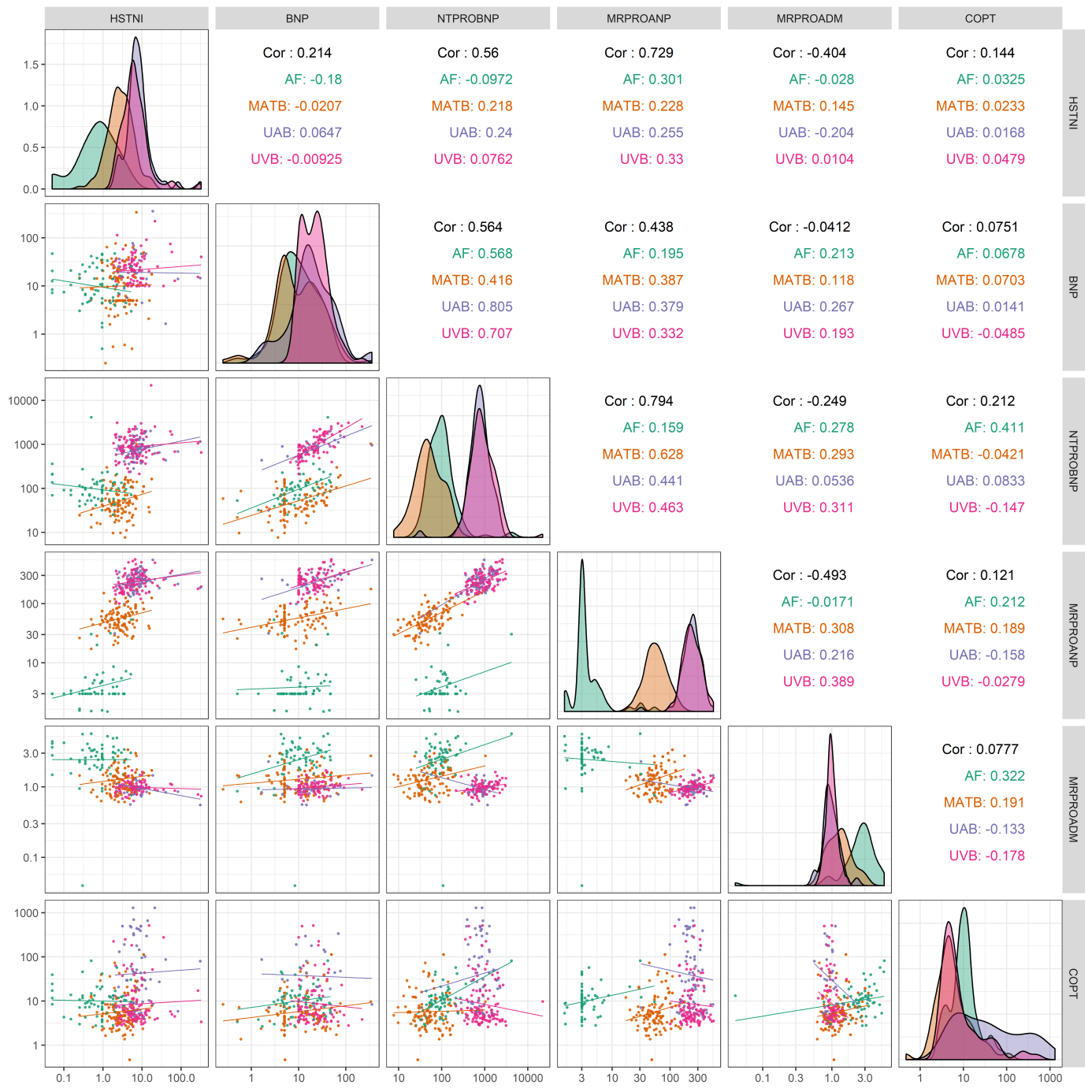


Supplement 7: Correlation matrix for biomarkers in elective cesarean section. The correlation matrix shows bivariate correlations between the analyzed six different biomarkers in four different body fluid as scatterplots and correlation coefficients (Spearman's rho). The superimposed curves shown in the diagonal center axis represent frequency curves for individual values in the individual body fluids. The four examined body fluids are color coded: AF (amniotic fluid) green; MATB (maternal blood) orange; UAB (umbilical arterial blood) blue; UVB (umbilical venous blood) purple. Examined biomarkers were high sensitive troponin I (hsTnI [pg/mL]), brain natriuretic peptide (BNP [pg/mL], N-terminal-pro brain natriuretic peptide (NTproBNP [pg/mL]), midregional pro-atrial natriuretic peptide (MRproANP [pmol/L]),), midregional pro-adrenomedullin (MRproADM [nmol/L]); copeptin (COPT [pmol/L]). The database consisted of n=129 neonates and their mothers.
